# Design and Evaluation of Novel Polymyxin Fluorescent Probes

**DOI:** 10.3390/s17112598

**Published:** 2017-11-11

**Authors:** Bo Yun, Kade D. Roberts, Philip E. Thompson, Roger L. Nation, Tony Velkov, Jian Li

**Affiliations:** 1 Drug Delivery, Disposition and Dynamics, Monash University, Parkville, Victoria 3052, Australia; Bo.Yun@petermac.org (B.Y.); Kade.Roberts@monash.edu (K.D.R.); roger.nation@monash.edu (R.L.N.); 2 Medicinal Chemistry, Monash Institute of Pharmaceutical Sciences, Monash University, Parkville, Victoria 3052, Australia; Philip.Thompson@monash.edu; 3 Monash Biomedicine Discovery Institute, Department of Microbiology, Monash University, Clayton, Victoria 3800, Australia

**Keywords:** polymyxins, fluorescent, dansyl, probe, gram-negative

## Abstract

Polymyxins (polymyxin B and colistin) are cyclic lipopeptide antibiotics that serve as a last-line defence against Gram-negative “superbugs”. In the present study, two novel fluorescent polymyxin probes were designed through regio-selective modifications of the polymyxin B core structure at the *N*-terminus and the hydrophobic motif at positions 6 and 7. The resulting probes, FADDI-285 and FADDI-286 demonstrated comparable antibacterial activity (MICs 2–8 mg/L) to polymyxin B and colistin (MICs 0.5–8 mg/L) against a panel of gram-negative clinical isolates of *Acinetobacter baumannii*, *Klebsiella pneumoniae* and *Pseudomonas aeruginosa*. These probes should prove to be of considerable utility for imaging cellular uptake and mechanistic investigations of these important last-line antibiotics.

## 1. Introduction

Over the past two decades there has been a pronounced increase in the emergence of multidrug-resistant (MDR) Gram-negative “superbugs”, leading to serious infections that are resistant to almost all currently available antibiotics [1]. The dire situation is perpetuated by a lack of novel antibiotics in the developmental pipeline, leaving the world in a vulnerable state against these life-threatening infections [1]. This “perfect storm” has led to the revival of the polymyxin class of antibiotics, polymyxin B and E (the latter also known as colistin), as a last line of defence against MDR Gram-negative “superbugs” [2]. However, despite their excellent antibacterial activity, the use of polymyxins has largely been limited by a high incidence of nephrotoxicity among patients receiving these antibiotics [3,4,5,6]. 

**Figure 1:**
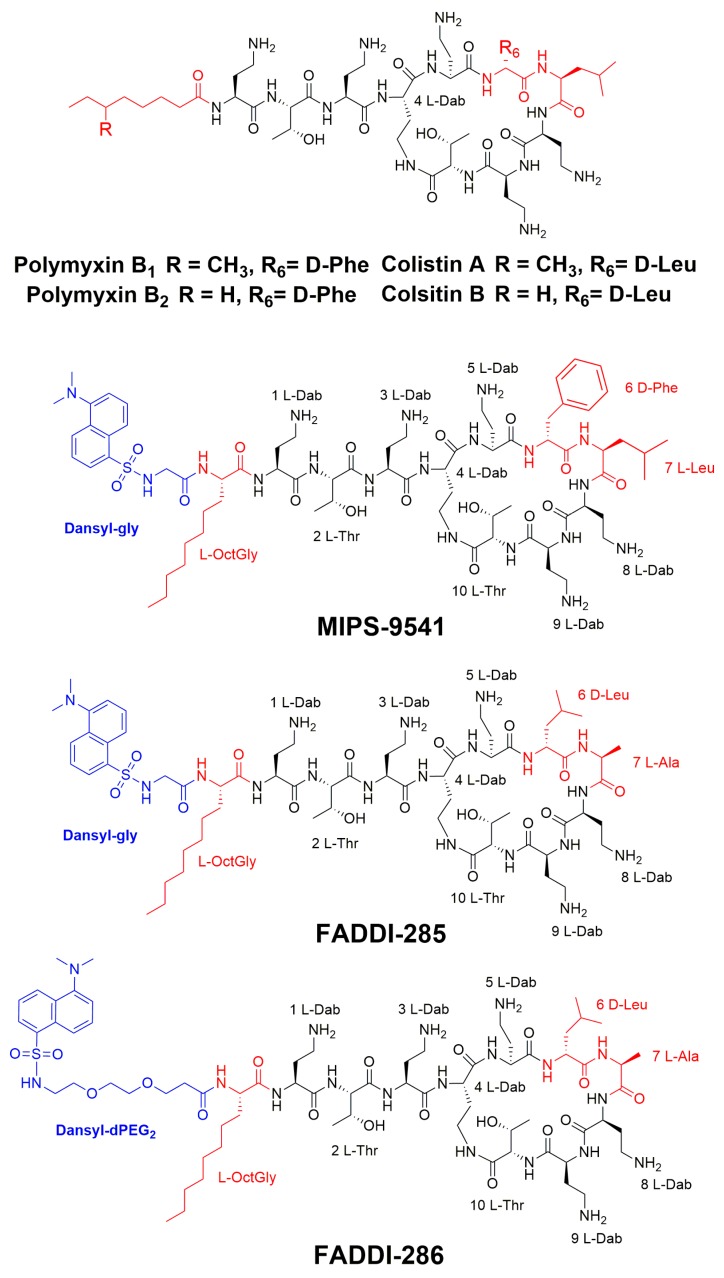
Structures of polymyxin B, colistin, MIPS-9541 and the novel fluorescent polymyxin probes FADDI-285 and FADDI-286.

Polymyxins are amphipathic cationic lipopeptides, comprised of hydrophobic and hydrophilic domains that are critical for their antibacterial activity [7]. The general polymyxin structure consists of a cyclic heptapeptide ring with a linear tripeptide segment and an *N*-terminal fatty acyl tail (Figure 1). Additionally, there are five L-α,γ-diaminobutyric acid (Dab) residues, which contain primary amines that are positively charged at physiological pH (7.4), as well as two hydrophobic residues in positions 6 and 7 of the cyclic ring. The two polymyxins used clinically, polymyxin B and colistin, are differentiated by a single hydrophobic residue at position 6: D-leucine in colistin and D-phenylalanine in polymyxin B [7]. Both polymyxins are products of fermentation and are mixtures, each containing two major components, colistin A and B and polymyxin B_1_ and B_2_, which differ by one carbon at the fatty acyl tail (Figure 1). The fatty acyl tail is essential for the antibacterial activity of polymyxins, since polymyxin nonapeptide (PMBN) (produced by proteolytic removal of the fatty acyl-Dab1 from the *N*-terminus of the polymyxin) is inactive [8,9,10]. These structural features of the polymyxin core scaffold are critical for interaction with the initial target, lipid A of the outer membrane.

Commercial probes (e.g., dansyl- and BODIPY-polymyxin B) have been utilized in polymyxin mechanistic studies, however, they lack antimicrobial activity due to the blockage of multiple Dab residues (potentially up to all five); therefore, these compounds are not structurally representative of the parent polymyxin [11]. Our group has previously reported the design and synthesis of regio-selectively mono-dansylated polymyxin B probes such as MIPS-9541 (Figure 1) for exploring polymyxin mechanisms of action and imaging of polymyxin interactions with kidney proximal tubular cells [12]. In the current study, we build on our novel design strategy generating the novel fluorescently labelled polymyxin probes, FADDI-285 and FADDI-286 (Figure 1) which are representative of the native polymyxins, and retain antimicrobial activity. These novel fluorescent polymyxin probes should have improved in vivo utility and help facilitate medicinal chemistry strategies to ameliorate unwanted nephrotoxicity and resistance that limit the clinical efficacy of these important last-line lipopeptide antibiotics. 

## 2. Methods

### 2.1. Chemical Reagents

Diisopropylethylamine (DIPEA) was obtained from Auspep (Melbourne, Australia). Fmoc-L-OctGly-OH and Fmoc-Dab(Boc)-OH were obtained from Try-lead Chem (Hangzhou, China). Fmoc-Dab(ivDde)-OH, Fmoc-D-Leu-OH, 1H-Benzotriazolium-1-[bis(dimethylamino)methylene]-5-chloro hexafluoro- phosphate-(1-),3-oxide (HCTU) and 1,1,1,3,3,3-Hexafluoro-2-propanol (HFIP) were obtained from Chem-Impex International (Wood Dale, IL, USA). Fmoc-Thr(tBu)-OH, Fmoc-Ala-OH and Fmoc-Gly-OH were obtained from Mimotopes (Melbourne, Australia). *N*-Fmoc-Amido-dPEG_2_-OH was obtained from Peptides International (Louisville, KY, USA). Dichloromethane (DCM), dimethylformamide (DMF), diethyl ether and acetonitrile were obtained from Merck (Melbourne, Australia). Fmoc-Thr(tBu)-TCP-Resin was obtained from Intavis Bioanyltical Instruments (Köln, Germany). Piperidine, triisopropylsilane (TIPS), trifluoroacetic acid (TFA), dansyl-chloride, ethanedithiol (EDT) and diphenylphosphorylazide (DPPA) were obtained from Sigma-Aldrich (Castle Hill, Australia) Polymyxin B sulfate and colistin sulfate were research grade and obtained from BetaPharma (Shanghai, China). 

### 2.2. HPLC Purification and LC-MS Analysis

Peptides were purified by RP-HPLC on a Waters Prep LC system incorporating a Waters 486 tuneable absorbance detector set at 214 nm and a Phenomenex Luna C8(2) column (250 × 21.2 mm ID, 100 Å, 10 micron). Peptides were eluted with a gradient of 100% Buffer A (0.1% TFA/water) to 60% Buffer B (0.1%TFA/acetonitrile) over 60 min at a flow rate of 15 mL/min. Fractions collected were analysed by LC/MS on a Shimadzu 2020 LCMS system. LC analysis was carried out at 214 nm using a Phenomenex Luna C8(2) column (100 × 2.0 mm ID, 100 Å, 3 micron), eluting with a gradient of 100% Buffer A (0.05% TFA/water) to 60% Buffer B (0.05%TFA/acetonitrile) over 10 min at a flow rate of 0.2 mL/min. Mass spectra were acquired in positive ion mode with a scan range of 200–2000 m/z. 

### 2.3. Synthesis FADDI-285 

Synthesis of the protected linear peptide precursor was conducted on a Protein Technologies Prelude automated peptide synthesizer using pre-loaded Fmoc-Thr(tBu)-TCP resin (0.1 mmol scale). Fmoc deprotection was conducted using 20% piperidine in dimethylformamide (1 × 5 min, 1 × 10 min) at room temperature. Coupling of the Fmoc-amino acids for 50 min at room temperature using 3 molar equivalents of the Fmoc-amino acid and HCTU in DMF activated in situ, using 6 molar equivalents of DIPEA. The *N*-terminal dansyl group was coupled using 3 molar equivalents of dansyl-chloride in DMF in the presence of 6 molar equivalents of DIPEA for 50 min at room temperature. The resin was then treated with 3% Hydrazine/ DMF (4 × 15 min) to remove the ivDde group. The protected linear peptide was then cleaved from the resin by treating the resin with 10% HFIP in DCM (1 × 30 min, 1 × 5 min). This solution was concentrated *in vacuo* to produce the protected linear peptide as a residue. The protected linear peptide was dissolved in DMF (10 mL) to which DPPA 0.3 mmol, 0.65 µL (3 molar equivalents relative to the loading of the resin) and DIPEA 0.6 mmol, 104 µL (6 molar equivalents relative to the loading of the resin) were added. This solution was stirred at room temperature overnight. The reaction solution was then concentrated under vacuum overnight to give the crude protected cyclic peptide as a residue. The resulting residue was taken up in a solution of 2.5% EDT/5% TIPS/TFA and shaken at room temperature for 2 h. To this solution 40 mL of diethyl ether was added. The resulting precipitate was collected by centrifugation and washed twice more with diethyl ether (40 mL) then air-dried in a fume hood to give the crude cyclic peptide as a solid. The resulting solid was taken up in Milli-Q water (5 mL) and de-salted using a Vari-Pure IPE SAX column. The eluent containing the crude cyclic peptide was acidified with TFA (10 L) and subjected to RP-HPLC purification as described above. Fractions collected were analysed by LC-MS as described above. Fractions containing the desired product were freeze-dried to give the **FADDI-285** TFA salt as a pale-yellow solid in a yield of 57.2 mg (>95% purity). Molecular weight was confirmed by ESI-MS analysis; m/z (monoisotopic) calculated: C_65_H_111_N_19_O_16_S 1446.82, [M + 2H]^2+^ 723.91, [M + 3H]^3+^ 482.93; observed: [M + 2H]^2+^ 724.30. [M + 3H]^3+^ 483.50.

### 2.4. Synthesis FADDI-286 

This peptide was synthesized as described above for **FADDI-285** to give the **FADDI-286** TFA salt as a pale-yellow solid in a yield of 65.2 mg (>95% purity). Molecular weight was confirmed by ESI-MS analysis; m/z (monoisotopic) calculated: C_70_H_121_N_19_O_18_S [M + H]^+^ 1548.89, [M + 2H]^2+^ 774.94, [M + 3H]^3+^ 516.96; observed: [M + 2H]^2+^ 775.40, [M+3H]^3+^ 517.60.

### 2.5. Determination of MICs

MICs against *Pseudomonas aeruginosa*, *Klebsiella pneumoniae*, and *Acinetobacter baumannii* strains were determined by the broth microdilution method (CLSI 2013). Experiments were conducted in 96-well polypropylene microtitre plates with all dilutions using cation-adjusted Mueller-Hinton broth (CaMHB). Bacterial suspension (100 µL, containing ~10^6^ colony forming units (CFU) per mL) was added to the wells in the presence of increasing concentrations of polymyxins (0 to 128 mg/L). MICs are defined as the lowest concentration at which visible growth was inhibited after overnight incubation at 37 °C. 

## 3. Results

### 3.1. Probe Design and Synthesis

**Figure 2:**
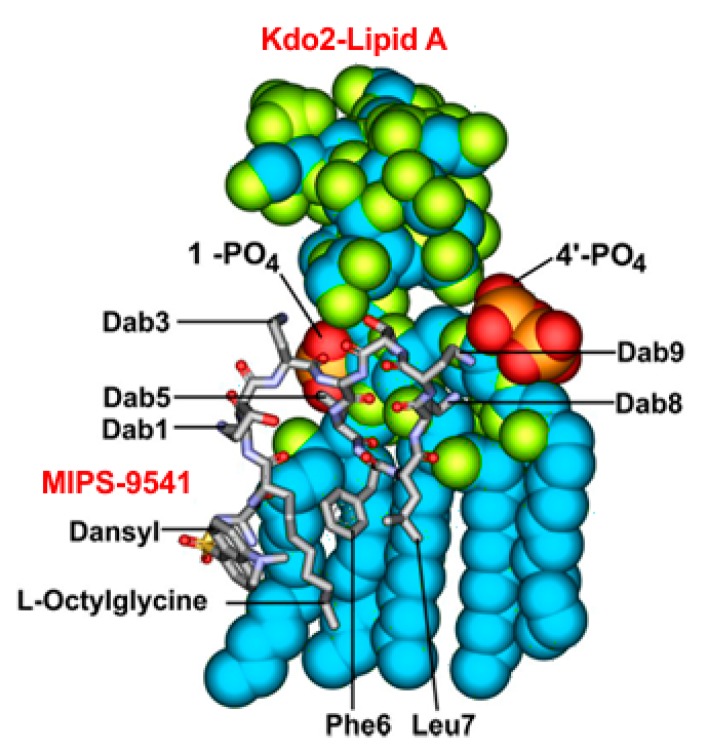
NMR derived model of MIPS-9541 in complex with bacterial Kdo2-lipid.

Previously we designed the regio-selectively mono-dansylated probe MIPS-9541 in which the *N*-terminal fatty acyl group of polymyxin B was substituted with dansylglycine-octanylglycine (Figure 1) [11]. The hydrophobic dansyl group was utilized as the fluorophore, as its comparatively small size relative to other fluorophores would help to reduce the likelihood of negative steric effects on the polymyxin pharmacophore [7]. The L-octylglycine (C8) residue serves to emulate the eight carbon *N*-terminal fatty acyl chain of the polymyxins and also provides a point for attachment of the dansyl fluorophore [11]. It has been previously demonstrated that a variety of hydrophobic groups are well tolerated at this position and act as mimics of the *N*-terminal saturated alkyl fatty acyl chains of polymyxin B [7]. NMR analysis showed that this probe has a similar mode of binding to lipid A, the initial target of the polymyxins such as the native polymyxin B (Figure 2). One of the concerns with the design of MIPS-9541, was the increase in the overall hydrophobicity of the scaffold resulting from the addition of the dansyl fluorophore which may have a negative effect on the ability of the molecule to closely mimic the native polymyxins. For example, increased hydrophobicity could lead to increased plasma protein binding leading to poor bio-distribution compared to the native polymyxins, which would have a negative impact on in vivo studies utilizing the probe [13]. In the current work, we have made further modification to MIPS-9541 in order to balance the hydrophobicity of the scaffold and improve its in vivo utility. To this end we incorporated a D-leucine residue at position 6 in place of D-Phe as seen in colistin and reduced the hydrophobicity at position 7 by substituting the leucine residue with a less hydrophobic alanine residue. This resulted in the generation of FADDI-285 (Figure 1). Previously it had been shown that the leucine residue at position 7 could be substituted with an alanine residue without loss of antibacterial activity against *P. aeruginosa* [14]. Further to these modifications, the glycine linker between the dansyl fluorophre and octylglycine residue was replaced with a PEG linker to generate FADDI-286. This PEG linker would help to decrease the hydrophobicity of the dansyl-octylglycine *N*-terminal modification. 

FADDI-285 and FADDI-286 had to be prepared using a total synthesis approach and were readily synthesized using standard solid-phase peptide synthesis and commercially available chemical reagents. Pleasingly, the modifications made to the polymyxin structure did not have a negative impact on the key cyclisation step to generate the heptapeptide cyclic ring, with final products being obtained in good yield and purity. 

### 3.2. Antibacterial Activity of the Probes

Antibacterial activity of both probes was assessed against the American Type Culture Collection (ATCC) strains and a panel of clinical isolates of polymyxin-susceptible *P. aeruginosa*, *K. pneumoniae* and *A. baumannii* (Table 1). Both probes demonstrated antibacterial activity against the polymyxin-susceptible strains (MICs 2–8 mg/L), comparable to polymyxin B and colistin (MICs 0.5–8 mg/L). Notably, both probes exhibited enhanced activity (MICs ~ 32 mg/L) against the polymyxin-resistant isolates (polymyxin B and colistin MICs ~ 128 mg/L).

## 4. Discussion and Conclusions

While bacteria have developed resistance to almost all other antibiotics, colistin and polymyxin B remain at the forefront of last-line therapeutics against MDR Gram-negative “superbugs” [15]. The primary focus of this study was to design and synthesize novel polymyxin fluorescent probes with the antibacterial activity representative of the native polymyxins. 

Polymyxins elicit their antimicrobial activity by first binding to the lipopolysaccharide in the bacterial outer membrane. The formation of the complex is initiated through the electrostatic interaction of the cationic Dab side-chains with the negatively charged phosphate groups of the lipid a component of LPS. This in turn displaces divalent cations (Ca^2+^ and Mg^2+^) that bridge adjacent LPS molecules, thereby de-stabilizing the outer membrane [2,7,16]. Subsequently, hydrophobic interactions occur between the *N*-terminal fatty acyl tail and the positions 6 and 7 hydrophobic segment of the polymyxin molecule and the fatty acyl chains of lipid A. The physical integrity of the phospholipid bilayer in the inner membrane appears to be subsequently disrupted [16,17,18,19,20]. This ‘self-promoted’ uptake mechanism is believed to lead to disruption of the cell envelope and bacterial killing [7,17,18,21]. With these principles in mind, we rationally designed the probes FADDI-285 and FADDI-286 via regio-selective modifications at the hydrophobic *N*-terminus and position 7, without markedly compromising antimicrobial activity of the parent colistin compound. Our group has previously highlighted the pitfalls of directly coupling fluorescent groups such as dansyl directly onto the Dab side chains in semi-synthetic preparations of dansyl-polymyxin B [11]. Furthermore, as polymyxin B and colistin are each comprised of two major components (polymyxin B_1_ and B_2_; colistin A and B), there is a strong possibility that either of these components will be substituted at any of the five Dab side chains resulting in a highly heterogeneous mixture of dansylated derivatives [11]. Indeed our previously reported mass spectrometry analysis of these semi-synthetic dansyl-polymyxin B preparations indicated the presence of a heterogeneous mixture of *mono*-, *di*-, and *tri*-dansyl Dab-substituted polymyxin B [11]. Accordingly, there is little value in using these semi-synthetic preparations as probes for imaging localization, since they lack the native antibacterial activity of the polymyxin B parent molecule and resultant images would represent the localization of a very complex array of probe molecules. Both FADDI-285 and FADDI-286 possess antibacterial activity similar to colistin and polymyxin B against polymyxin-susceptible isolates and notably, 4-fold enhanced activity against the polymyxin-resistant isolates (Table 1), and as such should prove to be of considerable utility as tools for novel polymyxin lipopeptide discovery programs. The utility of these probes in vivo is being investigated and will be the subject of a future report.

## Figures and Tables

**Table 1:** Minimum inhibitory concentrations (MICs) of each dansylated probe, polymyxin B and colistin against Gram-negative bacteria.

	*Pseudomonas aeruginosa* ATCC 27853	*Pseudomonas aeruginosa* FADDI-PA022	*Pseudomonas aeruginosa* FADDI-PA025	*Pseudomonas aeruginosa* FADDI-PA070	*Pseudomonas aeruginosa* FADDI-PA060	*Pseudomonas aeruginosa* FADI-PA090	*Acinetobacter baumannii* ATCC 19606	*Acinetobacter baumannii* FADDI-AB034	*Acinetobacter baumannii* ATCC 17978	*Acinetobacter baumannii* ATCC 19606 Col 10	*Acinetobacter baumannii* FADDI-AB156	*Acinetobacter baumannii* FADDI-AB167	*Klebsiella pneumoniae* ATCC 13883	*Klebsiella pneumoniae* FADDI-KP027	*Klebsiella pneumoniae* FADDI-KP003	*Klebsiella pneumoniae* FADDI-KP012
Peptide	MIC (mg/L)															
Colistin	1	1	2	>128	>128	8	1	0.5	0.5	128	16	8	1	>128	128	32
PolymyxinB	1	1	1	32	>32	4	1	0.5	1	128	8	16	1	128	>32	16
FADDI-285	2	2	4	>32	2	8	4	4	8	>32	8	8	4	>32	>32	>32
FADDI-286	2	2	4	>32	2	4	4	4	4	>32	8	8	4	>32	>32	>32
